# Integrating Clopidogrel’s First-Pass Effect in a Joint Semi-Physiological Population Pharmacokinetic Model of the Drug and Its Inactive Carboxylic Acid Metabolite

**DOI:** 10.3390/pharmaceutics16050685

**Published:** 2024-05-20

**Authors:** Zorica Pejčić, Valentina Topić Vučenović, Branislava Miljković, Katarina M. Vučićević

**Affiliations:** 1 Medicines and Medical Devices Agency of Serbia, Vojvode Stepe 458, 11221 Belgrade, Serbia; pejcic.z@gmail.com; 2 Department of Pharmacokinetics and Clinical Pharmacy, Faculty of Pharmacy, University of Belgrade, Vojvode Stepe 450, 11221 Belgrade, Serbia; branislava.miljkovic@pharmacy.bg.ac.rs; 3 Department of Pharmacokinetics and Clinical Pharmacy, Faculty of Medicine, University of Banja Luka, Save Mrkalja 14, 78000 Banja Luka, Bosnia and Herzegovina; valentina.topic.vucenovic@med.unibl.org

**Keywords:** clopidogrel, absorption, transit model, FPE, metabolism, PopPK, NONMEM

## Abstract

Clopidogrel (CLO), a pro-drug for preventing thrombotic events, undergoes rapid absorption and extensive metabolism, with approximately 85–90% converted to an inactive carboxylic acid metabolite (CLO-CA) and the remaining to an active thiol (CLO-TH). Few pharmacokinetic models for the drug and its metabolites exist, with most focusing on CLO-TH. Although CLO-CA is inactive, its predominant (compared to its parent drug and metabolites) presence in plasma underscores the importance of characterizing its formation and pharmacokinetic profile. This study aimed to characterize the process of the absorption of CLO and its conversion to CLO-CA via developing a population pharmacokinetic model. Individual participants’ data from two bioequivalence studies were utilized. Extensive blood samples were collected at predetermined intervals, including 841 concentrations of CLO and 1149 of CLO-CA. A nonlinear, mixed-effects modelling approach using NONMEM^®^ software (v 7.5) was applied. A one-compartment model was chosen for CLO, while a two-compartment proved optimal for CLO-CA. Absorption from the depot compartment was modeled via two transit compartments, incorporating transit rate constants (*K_tr_*). A semi-physiological model explained the first-pass effect of CLO, integrating a liver compartment. The estimated mean transit times (*MTTs*) for the studies were 0.470 and 0.410 h, respectively. The relative bioavailability for each study’s generic medicine compared to the reference were 1.08 and 0.960, respectively. Based on the estimated parameters, the fractions metabolized to inactive metabolites (*F_iaM__st1* and *F_iaM__st2*) were determined to be 87.27% and 86.87% for the two studies, respectively. The appropriateness of the final model was confirmed. Our model offers a robust framework for elucidating the pharmacokinetic profiles of CLO and CLO-CA.

## 1. Introduction

Clopidogrel (CLO) is a pro-drug that inhibits platelet aggregation and is indicated for the prevention of atherothrombotic and thromboembolic events in clinical conditions such as myocardial infarction, ischemic stroke, acute coronary syndrome, atrial fibrillation, and peripheral vascular diseases. The absorption of CLO after oral administration is rapid, with the maximum plasma concentration being reached after 45 min and at least 50% of the administered dose being absorbed. Clopidogrel is extensively metabolized in the liver. Most of the absorbed CLO (approximately 85% of an oral dose) is hydrolyzed by esterase to the inactive metabolite clopidogrel carboxylic acid (CLO-CA). Approximately 10–15% of orally absorbed CLO is oxidized in two steps by cytochrome P450 (CYP) isoenzymes to its active metabolite clopidogrel thiol (CLO-TH) [1,2,3,4]. In the first oxidation step, which is catalyzed by CYP1A2, CYP2B6 and CYP2C19, an intermediate metabolite, 2-oxo-CLO, is formed. In the second step, approximately 50% of the 2-oxo-CLO is further oxidized by CYP2B6, CYP2C9, CYP2C19 and CYP3A4/A5 to CLO-TH, which is responsible for the pharmacological effect of the drug. CLO-CA is the major circulating metabolite, with much higher plasma levels than both CLO and CLO-TH [1,2,5].

At the time of the development of the first CLO products, the pharmacokinetic profile of CLO was based on the inactive metabolite CLO-CA, as bioanalytical methods were not sensitive enough to quantify the concentrations of CLO or the active metabolite CLO-TH [6,7,8]. The introduction of state-of-the-art sensitive analytical methods has made it possible to reliably quantify CLO concentrations. Therefore, the current regulatory guidelines recommend that in the development of generic medicines, the demonstration of bioequivalence between generic and reference CLO medicines is based on the parent pro-drug [6]. Consequently, bioequivalence studies based on CLO [9,10], CLO-CA concentrations [11] or both [12,13,14,15,16] are available in the literature. Bioequivalence studies are designed to include frequent blood sampling, strictly standardized conditions and clearly defined inclusion/exclusion criteria for subjects to allow for good control of all potential sources of variability. A non-compartmental analysis is commonly acknowledged as the gold standard for determining bioequivalence, notwithstanding its’ constraints and the availability of compartmental and nonlinear, mixed-effects modeling approaches. The pharmacokinetic data obtained in bioequivalence studies are rich, especially during the absorption phase, and could therefore be used to characterize the absorption process including the first-pass effect (FPE) of the drug in more detail. Moreover, if both parent drug and metabolite concentrations are available, a joint pharmacokinetic model can be developed. For drugs that exhibit high inter-individual and intra-individual pharmacokinetic variabilities, such as CLO, a nonlinear, mixed-effects modeling approach may be more appropriate for delineating and quantifying the various levels of variability [17,18].

To the best of our knowledge, there are only a few published population pharmacokinetic models describing CLO and its metabolites [4,19,20,21,22,23,24], most of which focus on the CLO-TH metabolite in combination with the parent drug [4,22,23,24] or alone [21]. Physiologically based pharmacokinetic models have also been developed [3,25,26,27]. Although the metabolite CLO-CA is inactive, its predominant (compared to its parent drug and metabolites) presence in plasma underscores the importance of characterizing its formation and pharmacokinetic profile. To date, this inactive metabolite’s population pharmacokinetics models have been investigated in only a few publications, including a single joint-population pharmacokinetic model with its parent drug [4,19,20]. Hence, the aim of this study was to synthesize data from two bioequivalence studies in order to characterize the process of the absorption of CLO and its conversion to CLO-CA through a joint population semi-physiological pharmacokinetic model.

## 2. Materials and Methods

### 2.1. Subjects and Data

Individual participants’ data from two bioequivalence studies of CLO formulations, namely Study 1 and Study 2, were utilized. In each study, a single generic 75 mg film-coated tablet of CLO was assessed against Plavix 75 mg film-coated tablets (Sanofi Winthrop Industrie, Ambares, France), serving as the reference medicine. Both studies followed the same 2-way cross-over design (2 treatments, 2 periods, and 2 sequences), with subjects receiving a single CLO dose of 150 mg (2 tablets) under fasting conditions. The studies were conducted at the Military Medical Academy, Belgrade, Republic of Serbia, in compliance with the Declaration of Helsinki [28], the requirements of Good Clinical Practice [29], and the regulatory guidelines for bioequivalence studies conduct [30]. Institutional review board approval from the Military Medical Academy was obtained for the study protocols and informed consent forms. Secondary use of the study data was authorized by the study sponsor.

The eligible subjects were healthy adult volunteers of Caucasian origin of both sexes, with 24 in Study 1 and 26 in Study 2, all of whom had signed an informed consent form and met the predefined inclusion and none of the exclusion criteria. These criteria controlled for various factors that could have potentially influenced the results of the study, leading to more reliable and interpretable results. Study 1 involved healthy subjects aged 21 to 53 years whose body mass indexes (BMIs) ranged from 19 to 26 kg/m^2^, while Study 2 participants were aged 19 to 42 years and had BMIs of 19 to 29 kg/m^2^. During each study, the subjects each received the generic medicine once and the reference medicine once, according to the randomization scheme. Extensive blood samples were taken at predefined time intervals. In Study 1, sampling was conducted up to 48 h post-medicine administration, with 14 blood samples collected per subject per period, resulting in a total of 672 samples. In Study 2, sampling was performed up to 36 h after medicine administration, with 17 samples per subject per period, yielding a total of 884 samples. Plasma was separated from blood, and concentrations of CLO and CLO-CA were determined in both studies using a validated HPLC-MS analytical method, with a lower limit of quantification (LLOQ) of 0.5 ng/mL for CLO and 0.1 µg/mL for CLO-CA. Our dataset consisted of all demographic and clinical assessment data, including biochemical parameters, randomization scheme, time of dose administration and blood sampling, as well as reported concentrations of CLO and CLO-CA.

### 2.2. Data Analysis

The dataset for this study encompassed various parameters, including the time of dosing, individual values of the CLO and CLO-CA concentrations, time of blood collection and each study’s details (sequence, period and formulation), as well as all demographic (sex, age, body weight, height and BMI) and biochemical parameters (total bilirubin, serum creatinine, aspartate transaminase (AST) and alanine transaminase (ALT)). A descriptive statistical analysis was employed to evaluate the demographic characteristics and biochemical parameters of all 50 subjects from both studies. A population pharmacokinetic analysis was conducted using a nonlinear mixed effects modelling approach with NONMEM^®^ software (v7.5, ICON development solutions Ellicott City, MD, USA) [17,31,32]. Descriptive statistics and graphical presentations of the results were created using the appropriate packages in R^®^ (v4.1.3, R Foundation for Statistical Computing) [33] and RStudio^®^ (v1.4.1717) [34].

A total of 841 non-zero concentrations of CLO and 1149 of CLO-CA were used in the pharmacokinetic modelling. Data below the LLOQ (BLLOQ) were excluded from the analysis. The process of pharmacokinetic model-building involved multiple steps, concentrating on both the structural and statistical parts for both CLO and CLO-CA. The absorption of CLO was evaluated using various models, starting from a simple first-order model without and with a delayed absorption onset. Furthermore, considerations were made for absorption via hard-coded (2, 3 or 4 compartments) and estimated numbers of transit compartments [35]. Both one- and two-compartment models with first-order elimination were explored for both CLO and CLO-CA.

Given that CLO demonstrates an extensive FPE, exceeding 90%, the conversion of CLO to CLO-CA was examined using a model that incorporated a liver compartment. It was presumed that all parent drugs would ultimately undergo metabolism in this compartment. Integrated semi-physiological models of similar structure with FPEs have been previously published [4,24,36,37]. The model employed a physiological liver volume of 1.5 L and a liver plasma flow (*Q_h_*) of 50 L/h [24]. To account for the differences in molecular weights between CLO and CLO-CA (321.82 and 307.80, respectively), a scaling factor of 0.9565 was applied to the formation rate of CLO-CA. The fractions of the dose metabolized to the inactive CLO-CA (*F_iaM_*), active metabolite CLO-TH (*F_aM_*) and remaining fraction of parent drug (*F_P_*) were assumed to follow a logit-normal distribution. Hence, they were study-specific and coded as follows:

(1) $$F_{iaM}=\frac{{FR}_{1}}{1+{FR}_{1}+{FR}_{2}},$$

(2) $$F_{aM}=\frac{{FR}_{2}}{1+{FR}_{1}+{FR}_{2}}\;and$$

(3) $$F_{P}=\frac{1}{1+{FR}_{1}+{FR}_{2}},$$

where *FR*_1_ and *FR*_2_ are model parameters supporting the calculation of the corresponding fractions of the dose without any physiological meaning. Certain parameters needed to be fixed to ensure the stability of the model. Hence, *F_aM_* was fixed to the estimated value of 12%, which corresponded to the literature data [1,2,3,4]. Nevertheless, a sensitivity analysis was performed by varying the values of *F_aM_* at 10% and 15%. *F_iaM_* and *F_P_* were calculated from the estimated *FR*_1_ in the model for each study. Initially, clearance of the parent drug (*CL_P_*) was estimated at 89.5 L/h, and subsequently, it was fixed at the estimated value in order to stabilize the model. Bioavailability for the reference medicine (*F*) was fixed at 100%, and the relative bioavailability for the generic medicines from each of the two studies (*F_gen__st1* and *F_gen__st2*) were estimated during modeling.

Inter-individual (IIV) random effects were described by a log-normal distribution of the parameters while a study-specific proportional error model described the intra-individual variability. Moreover, considering the cross-over design of bioequivalence studies, inter-occasional variability (IOV) was incorporated in the absorption parameters.

Classical covariate model-building was not applied, as the inclusion/exclusion criteria ensured a homogenous population of healthy subjects, and there were no differences in the demographic characteristics and biochemical parameters among the studies. However, the incorporation of body-weight via allometric scaling was applied to corresponding clearance(s) using an allometric exponent of 0.75 and volume(s) of distribution with an exponent of 1.

The adequacy of the model was determined based on several criteria, including successful minimization, number of significant digits, gradients in the last iteration, values and changes in objective function (OFV), Akaike information criterion (AIC), Bayesian information criterion (BIC) and precision of parameter estimates. These factors collectively assessed the reliability and accuracy of the model in capturing the observed data and its ability to provide meaningful insights into the underlying pharmacokinetic behavior based on the population semi-physiological model. Furthermore, a graphical evaluation employing goodness-of-fit plots was used to assess the agreement between the observed and predicted values of the CLO and CLO-CA concentrations across the different time points. These diagnostics provided insights into the model’s predictive performance [31,38,39].

The nonparametric sample importance resampling (SIR) technique, including 1000 replicates, was employed [40]. The parameters’ 95% confidence intervals (CI), obtained through SIR-based simulations, were compared to the estimates derived from the dataset. Additionally, the predicted performance of the final model was assessed using a visual predictive check [41,42], involving 1000 simulations. By plotting the observed data alongside the simulated data from the model, the VPCs allowed for a visual comparison of how well the model predicted the central tendencies and variabilities in the observed data.

## 3. Results

The demographic characteristics and biochemical parameters of a total 50 subjects from two bioequivalence studies are presented in Table 1. There were no statistically significant differences observed in the subjects’ characteristics among the studies.

**Figure 1:**
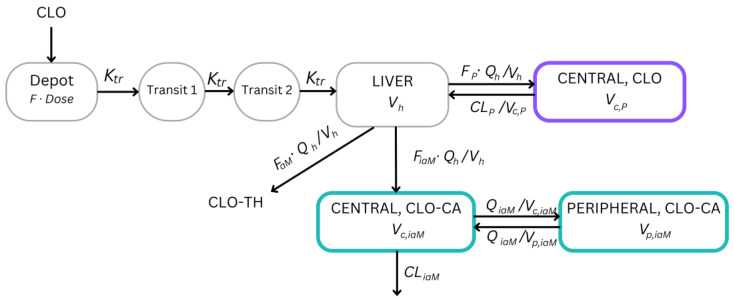
A schematic illustration of the final joint population semi-physiological pharmacokinetic model for clopidogrel (CLO) and its active (CLO-TH) and inactive (clopidogrel carboxylic acid (CLO-CA)) metabolites. *CL_iaM_*, clearance of inactive metabolite; *CL_P_*, clearance of parent drug; *F*, bioavailability; *F_aM_*, fraction metabolized to active metabolite; *F_iaM_*, fraction metabolized to inactive metabolite; *F_P_*, remaining fraction of the parent drug; *K_tr_*, transit rate constant; *Q_h_*, liver plasma flow; *Q_iaM_*, intercompartmental clearance of inactive metabolite; *V_c_*_,*iaM*_, volume of central compartment of inactive metabolite; *V_p_*_,*iaM*_, volume of peripheral compartment of inactive metabolite; *V_c_*_,*P*_, volume of distribution of parent drug; *V_h_*, volume of the hepatic (liver) compartment.

The final dataset for the pharmacokinetic analysis consisted of 841 non-zero concentrations of CLO and 1149 of CLO-CA. The BLLOQs for CLO and CLO-CA were excluded from the analysis. A majority (87.48%) of the excluded BLLOQs were at/after 6 h for CLO, and 78.50% of those were at/after 12 h for CLO-CA. The maximum plasma concentrations were, on average, 7.2 ng/mL and 4.9 µg/mL for CLO and CLO-CA, respectively. Due to issues with parameter unidentifiability when using the two-compartment model, we opted to utilize a one-compartment model for CLO instead. Conversely, for CLO-CA, the best fit was achieved with the two-compartment model. Absorption from the depot compartment was best described with a transit model including two transit compartments with a transit rate constant (*K_tr_*). The mean transit time (*MTT*) was estimated for each study. As previously described, a hepatic (liver) compartment was utilized to characterize the FPE of CLO. A schematic illustration depicting the structural model for CLO and CLO-CA is presented in Figure 1.

Ultimately, the IIV exponential model was incorporated in the volume of distribution of the parent drug (*V_c_*_,*P*_), the volume of the central compartment for the inactive metabolite (*V_c_*_,*iaM*_) and *FR_1_* and *F* for each study, separately. When added to the other parameters, IIV resulted in imprecise estimates and over-parameterization of the model. Exponential models ensure positive values for individual parameters, and consequently, they are the most preferred model for IIV. The inclusion of IOV on the absorption parameters *F* and *MTT* led to a statistically significant reduction in OFV by 284.202 and in AIC by 264.562. AIC as well as BIC values serve as alternatives to OFV for assessing how well a model fits its data while also factoring in its complexity. A lower AIC value suggests a better balance between model fit and its complexity. BIC, on the other hand, leans towards simpler models with fewer parameters while also considering the sample size for assessing model complexity. Hence, statistically significant drops in OFV, AIC and BIC values of 440.102, 420.102 and 364.143, respectively, were observed starting from the base to the final model. A total of 24 (including 11 structural) model parameters were estimated (Table 2). The estimated *MTT* values for the two studies were 0.470 and 0.410 h, respectively. The relative bioavailability for each generic medicine compared to the reference medicine (*F_gen__st1* and *F_gen__st2*) were 1.08 and 0.960, respectively, and their 95% CIs included one. Based on the estimated parameters, the fractions metabolized to inactive metabolites (*F_iaM__st1* and *F_iaM__st2*) were determined to be 87.27% and 86.87% for the two studies, respectively. A summary of the CLO and CLO-CA parameter estimates obtained from the final population semi-physiological pharmacokinetic model is provided in Table 2. The relative standard errors for the structural parameters ranged up to 14.9%.

**Figure 2:**
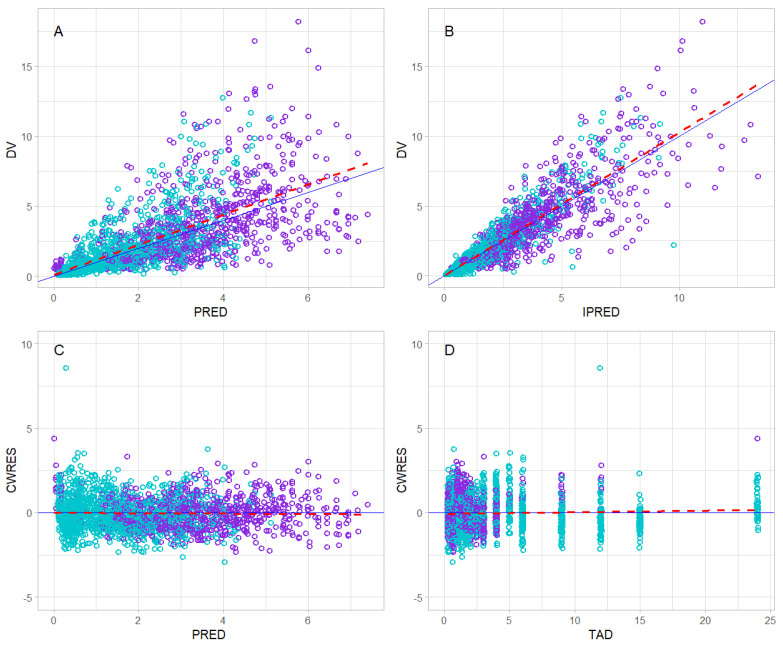
Goodness-of-fit plots of the final model for clopidogrel (CLO), shown in the purple color, and its inactive metabolite, clopidogrel carboxylic acid (CLO-CA), shown in the green color. Data for CLO (ng/mL) and CLO-CA (µg/mL) are presented together. (**A**) The observed (DV) versus population predicted values (PRED); (**B**) DV versus individual model predicted values (IPRED); (**C**) conditional weighted residuals (CWRES) versus PRED; and (**D**) CWRES versus time after dose (hour). The solid lines represent the lines of identity in (**A**,**B**) and the zero lines in (**C**,**D**). The dashed lines represent the regression lines.

Diagnostic plots of the final model are presented in Figure 2. Data for the observed and model predicted concentrations of CLO and CLO-CA are presented together; however, the CLO concentration was measured in ng/mL while CLO-CA was measured in µg/mL.

**Figure 3:**
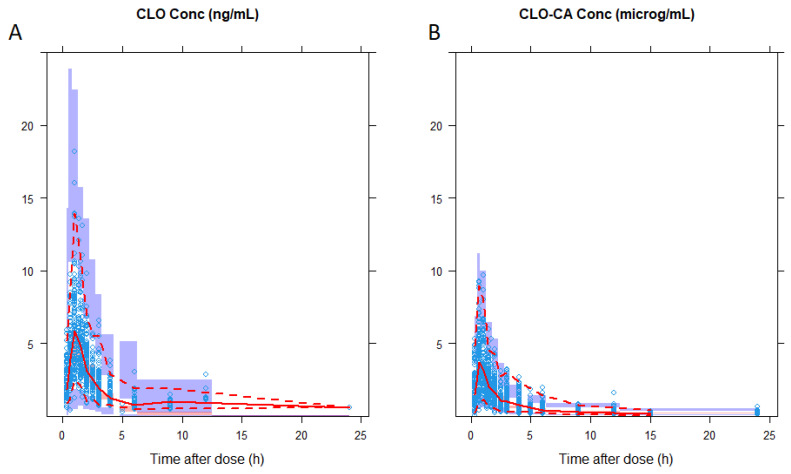
Visual predictive checks of the final joint population pharmacokinetic model for (**A**) clopidogrel (CLO) and (**B**) its inactive metabolite, clopidogrel carboxylic acid (CLO-CA). The observed concentrations are depicted by blue rectangles. The solid and dashed red lines represent the 50th, 5th and 95th percentiles of the predictions, respectively. The semi-transparent red shaded area represents the simulation-based 95% confidence interval (CI) for the median, while the semi-transparent purple fields represent the 95% CI around the 5th and 95th percentiles of the predicted data.

The appropriateness of the final population semi-physiological pharmacokinetic model and parameter precision evaluation was confirmed by the SIR technique. The typical parameter estimates, standard errors and 95% CIs using the original dataset were similar to those obtained from 1000 successful replicate runs (Table 2). Moreover, model appropriateness was visually confirmed via VPCs for both CLO and CLO-CA, which showed good agreement between the measured and simulated concentration data (Figure 3).

## 4. Discussion

We developed a joint population semi-physiological pharmacokinetic model for CLO and its inactive metabolite, CLO-CA, using nonlinear, mixed-effects modelling, leveraging data from two bioequivalence studies. The frequent blood-sampling conducted during these studies allowed for the comprehensive pharmaco-statistical characterization of CLO absorption and FPE. Out of 1556 blood samples collected per the protocols, a total of 841 non-zero CLO concentrations (54.04%) and 1149 non-zero CLO-CA concentrations (73.84%) were used in the analysis. Based on our analysis, the terminal half-life of CLO averaged 1.69 (1.46–1.92) hours. Consequently, on average, CLO was expected to completely eliminate after approximately 10 h, indicating that the sampling duration may have been too long. Indeed, a majority of the reported BLLOQs for CLO were at least 12 h after drug administration. This was comparable with the study by Jung et al. [4], where BLLOQ data for CLO were omitted, constituting 27% of the total, with blood-sampling times extending up to 24 h. Considering the sensitivity of the bioanalytical method used in Study 1 and Study 2, it is unsurprising that numerous concentrations of CLO fell into the BLLOQ category. Analogous considerations were applied to the BLLOQ data for CLO-CA, justifying the exclusion of BLLOQ data from the analysis. In each of the studies, the administration of two tablets (150 mg of CLO) was planned per the protocols as it would result in higher concentrations, and the bioanalytical method might have been sensitive enough to accurately quantify them. Therefore, our maximum concentrations and area under the concentration–time curves for both the parent drug and the metabolite exceeded what was expected after 75 mg according to the Summary of Product Characteristics [1].

For a comprehensive understanding of the complete pharmacokinetic profile of CLO, it is crucial to characterize not only its active metabolite CLO-TH, as documented in the literature [21,22,23,24], but also the inactive metabolite CLO-CA. CLO is rapidly metabolized, leading to CLO-CA being present in plasma at approximately 2000 times higher concentrations than the parent drug, as previously reported [1,6]. Moreover, studies have demonstrated a linear correlation between CLO and CLO-CA concentrations [43], and CLO-CA concentrations have been previously employed to investigate CLO pharmacokinetics during bioequivalence studies [6]. Furthermore, studies have reported that assessing CLO-CA plasma concentrations is clinically valuable for identifying poor compliance and variable metabolisms in patients receiving CLO therapy. This suggests that plasma concentrations of CLO-CA serve as reflections of the clinical efficacy of CLO [7]. Although there are limited studies on CLO absorption and FPE to inactive metabolite [4,19,20], it remains crucial to thoroughly describe this process. The models developed by Yousef et al. [19] and Lee et al. [20] were solely based on the concentrations of the inactive metabolite CLO-CA. Therefore, we conducted an in-depth analysis to enhance the understanding of CLO pharmacokinetics, complementing the findings of Jung et al. [4], who developed a comprehensive joint model incorporating both the inactive and active metabolites. However, unlike the authors who kept the fractions fixed at values obtained from the literature, we estimated them.

Despite combining data from two studies and aiming to develop a unified model for CLO, we opted to estimate certain parameters separately for each study (Table 2). This approach was adopted as the data were derived from different study conditions, and as it led to a decrease in OFV and AIC and/or BIC values and stabilized the model.

The final model we developed effectively captured the pharmacokinetic properties of CLO and its metabolite CLO-CA, as evidenced by the numerical and graphical validation. Our model incorporated a liver compartment (Figure 1), aligning with the well-documented and extensive CLO pre-systemic hepatic metabolism of 85–90% of the absorbed CLO [1,2,3,4]. Complete metabolism of the parent drug was supported by the return of CLO to the liver from the central compartment (Figure 1). Due to the availability of CLO and CLO-CA concentrations from each study, our aim was to indirectly estimate the fractions, *F_iaM_* and *F_P_*, per study, while fixing the fraction of CLO metabolized to CLO-TH at 12%. A sensitivity analysis was performed, and the model was robust (the OFV changed by fewer than two units) to the varying values of *F_aM_* of 10% and 15%. The obtained values for the fractions metabolized to CLO-CA were approximately 87% for both studies, aligning closely with the literature values of at least 85–90% [1,2,3,4]. This estimation resulted in a one-compartment model for CLO, as in Jiang et al. [24], in contrast to the two-compartment models in [4,23]. This difference was likely attributable to fixing the parameters used for the calculation of the metabolite fractions in Jung et al. [4], whereas we estimated *FR_1__st1* and *FR_1__st2* directly. CLO exhibits fast absorption, with a time to the maximum concentration of approximately 45 min [1]. Various absorption models have previously been used to characterize the process, including first-order absorption with/without lag time [4,19] and transit compartments [20,23,24]. Erlang-type absorption [44] with two transit compartments improved the absorption phase of CLO compared with the lag model (a statistically significant drop in the AIC of 5.416 units). This model provided a closer description of the absorption that was in line with the physiological conditions. Based on the estimated *MTT*s and two transit compartments, the transit rate constants (*K_tr_*) equaled 6.38 h^−1^ and 7.32 h^−1^ for each study, respectively. Overall, the closest in structure to ours was the model established by Jiang et al. [24], but they considered a parent and an active metabolite of CLO, estimating fractions entirely physiologically using well-stirred models for clearances.

The two-compartment model effectively described the CLO-CA concentration-time profile, and the parameters’ values were in agreement with previously reported data [19,20]. All final model parameters were estimated with adequate precision (Table 2). The diagnostic plots indicated good agreement between the population/individual predictions and the observed concentrations of CLO and CLO-CA (Figure 2A,B). The conditional weighted residuals (CWRES) were randomly scattered around zero across the population predictions and time after dose (Figure 2C,D). The VPCs, and shown in Figure 3, indicated the good predictive abilities of the model for both the parent drug and the inactive metabolite of CLO.

The model we developed lays a solid groundwork for potential future enhancements to create a more complex model. This could involve incorporating the formation of the active metabolite CLO-TH, genetic variations, and perhaps other factors (such as concurrent drug therapies that interact with CLO) gleaned from patient data.

Despite the strengths of our investigation, there are some limitations to consider. The two bioequivalence studies were independent and involved different subjects, although all were healthy adults with no significant differences in demographic characteristics and biochemical parameters. Therefore, a typical stepwise covariate modeling approach was not justified. This is why body-weight was allometrically included in the model. The impact of varying CYP2C19 and/or carboxylesterase 1 (CES1) phenotypes on CLO pharmacokinetics has been previously recognized [2,22,24,25,45,46,47]. However, polymorphism data were not available in the studies we used. Nevertheless, even if we had access to CYP2C19 polymorphism data, it is less likely that the covariate would enhance the model, given that CYP2C19 does not play a critical role in CLO-CA formation. On the contrary, as CES1 is responsible for the formation of the inactive metabolite [24,47], the availability of CES1 phenotypes may have resulted in its inclusion in our final model.

## 5. Conclusions

In conclusion, we have developed a comprehensive joint semi-physiological pharmacokinetic model for CLO and its inactive metabolite, CLO-CA. This model incorporates two transit compartments for absorption and accounts for FPE in a liver compartment. Our model is based on bioequivalence data obtained from two studies, providing a robust framework for understanding the pharmacokinetics of these compounds.

## Figures and Tables

**Table 1:** Demographic characteristics and biochemical parameters of the subjects.

Characteristics (Units)		Number (%)/Mean ± Standard Deviation (Range)
Gender	Male	29 (58.00)
	Female	21 (42.00)
Age (year)		31.94 ± 8.51 (19–54)
Body-weight (kg)		74.1 ± 13.56 (47–100)
Height (cm)		177.26 ± 9.06 (155–194)
Body mass index (kg/m^2^)		23.40 ± 2.66 (19.10–29.30)
Bilirubin (µmol/L)		9.1 ± 4.43 (3–25)
Serum creatinine (µmol/L)		81.5 ± 17.70 (53–114)
Alanine transaminase (ALT) (U/L)		26.2 ± 9.85 (11–52)
Aspartate transaminase (AST) (U/L)		23.7 ± 4.80 (16–35)

**Table 2:** Final parameter estimates for the joint population pharmacokinetic model for clopidogrel (CLO) and its inactive metabolite, clopidogrel carboxylic acid (CLO-CA).

Parameters (Units)	Dataset		Sampling Importance Resampling (SIR)	
	Estimate	95% CI	Median	2.5–97.5 Percentile
*CL_P_* (L/h/70 kg)	89.5 FIX	-	-	-
*V_c_*_,*P*_ (L/70 kg)	218	188–248	217	189–243
*MTT_st1* (h)	0.470	0.425–0.515	0.471	0.429–0.516
*F_gen__st1*	1.08	0.993–1.17	1.08	0.995–1.16
*FR_1__st1*	119	84.3–154	118	88.2–155
*CL_iaM_* (L/h/70 kg)	8.70	7.38–10.0	8.60	7.59–9.73
*V_c_*_,*iaM*_ (L/70 kg)	23.7	19.7–27.7	23.4	20.0–26.9
*Q_iaM_* (L/h/70 kg)	10.8	8.02–13.6	10.8	8.94–13.0
*V_p_*_,*iaM*_ (L/70 kg)	61.3	50.3–72.3	60.9	52.4–70.2
*Q_h_* (L/h)	50 FIX	-	-	-
*V_h_* (L/70 kg)	1.5 FIX	-	-	-
*MTT_st2* (h)	0.410	0.381–0.439	0.411	0.385–0.438
*F_gen__st2*	0.960	0.818–1.10	0.952	0.840–1.07
*FR_1__st2*	76.8	64.8–88.8	76.0	66.3–85.9
**Inter-Individual (IIV)/Inter-Occasional Variability (IOV)**	**Estimate CV (%)**	**RSE (%)**	**Median CV (%)**	**2.5–97.5 Percentile**
IIV *(V_c_*_,*P*_*)*	45.82	10.4	45.69	37.38–53.99
IIV *(V_c_*_,*iaM*_*)*	25.06	13.2	25.30	18.54–30.38
IIV *(F_st1)*	42.66	13.8	42.40	29.99–51.26
IIV *(F_st2)*	25.88	24.1	26.31	14.16–35.57
IOV *(F_st1)*	8.83	32.9	9.29	3.03–13.33
IOV *(F_st2)*	23.24	13.6	23.76	18.21–28.27
IOV *(MTT_st1)*	25.44	16.8	25.58	16.22–32.97
IOV *(MTT_st2)*	27.48	9.9	27.46	21.86–32.39
IIV *(FR_1__st1)*	72.80	10.5	73.59	57.52–89.97
IIV *(FR_1__st2)*	27.86	13.0	28.36	20.25–34.18
**Residual Error**	**Estimate (%)**	**RSE (%)**	**Median CV (%)**	**2.5–97.5 Percentile**
*Wp (st1)*	41.95	3.4	41.98	39.37–44.50
*Wp (st2)*	29.39	5.7	29.51	27.57–31.62

*CL_iaM_*, clearance of inactive metabolite; *CL_P_*, clearance of parent drug; CV, coefficient of variation; *FR_1__st1*, and *FR_1__st2*, fraction parameter for inactive metabolite for study 1and study 2, respectively; IIV, inter-individual variability for the corresponding parameter; IOV, inter-occasional variability for the corresponding parameter; *MTT_st1*, mean transit time for study 1; *MTT_st2*, mean transit time for study 2; *Q_h_*, liver plasma flow; *Q_iaM_*, intercompartmental clearance of inactive metabolite; *F_gen__st1* and *F_gen__st2,* relative bioavailability of generic compared to reference medicine for study 1 and study 2, respectively; RSE, relative standard error; *V_c_*_,*iaM*_, volume of central compartment of inactive metabolite; *V_h_*, volume of hepatic compartment; *V_p_*_,*iaM*_, volume of peripheral compartment of inactive metabolite; *V_c_*_,*P*_, volume of distribution of parent drug; *F_st1* and *F_st2*, bioavailability in study 1 and 2, respectively; *Wp*, proportional residual error for the corresponding study.

## Data Availability

The data are unavailable due to privacy restrictions.
